# Individualized Care and Follow-Up in Outpatient Surgery: A Pilot Study

**DOI:** 10.7759/cureus.33698

**Published:** 2023-01-12

**Authors:** Mafalda Martins, Inês Vaz, Helena Barbosa, Mariana Coroa, Alice Brás, Leonor Amaro

**Affiliations:** 1 Department of Anesthesiology, Centro Hospitalar Vila Nova de Gaia/Espinho, Vila Nova de Gaia, PRT

**Keywords:** economics of health care, quality improvement, patient-specific modeling, postoperative complications, continuity of patient care, cohort studies, ambulatory surgical procedures

## Abstract

Introduction

In outpatient surgery, post-discharge follow-up calls are essential for identifying complications and are considered a cost-effective intervention. Currently, there is a lack of scientific evidence to support the development and validation of standardized protocols adjusted to patients’ specificities. Our aim is to develop a personalized model for our outpatient surgery unit (OSU) to create an individualized follow-up strategy in the future.

Material and methods

We performed a retrospective, cohort, single-center study, including patients undergoing surgery at an OSU of a tertiary hospital in Portugal, for three months. Follow-up calls were performed on the seventh and fourteenth days after discharge. The variables analyzed included: sex, age, surgical specialty, anesthetic technique, American Society of Anesthesiologists (ASA) physical status classification, surgery duration, and complications. A binary logistic regression was adjusted for the complications detected in each call.

Results

Nine-hundred eighty-four (984) patients were included, of which 79.8% (n=785) and 75.3% (n=741) answered the follow-up calls on the seventh and fourteenth days after discharge, respectively. Complications were reported in 47.1% (n=370) and 29.8% (n=221) of these calls, respectively, with pain having the highest incidence rate (44.7% in the first call; 26.6% in the second). The type of anesthesia and surgical specialty were independent risk factors for complications (p<0.001). Each minute increase in surgery duration increased by 1.1% the odds of complications (95% confidence interval 1.003-1.018) in the first call. Compared with no anesthesiology involvement, general anesthesia, regional anesthesia, and monitored anesthetic care are 2.52, 2.04, and 1.75 times more likely to have complications detected in the first call and 3.21, 2.36, and 3.11 times more likely to have complications on the second (p<0.05 for all). A model that predicts the detection of complications in each call was created.

Discussion

Outpatient surgery may allow procedures to be carried out safely, efficiently, and cost-effectively. To optimize the outcomes, it is important to quantify results as a tool for honing our strategies. The present study recognized the influence of several variables in the incidence of post-discharge complications. Also, considering the complications reported, pain was the most frequent among the reports and should not be neglected. In our reality, no follow-up calls are routinely performed after the seventh day, and complications were reported in that period, meaning some patients probably should be accompanied for a longer period.

Conclusions

To ensure the quality of care and patient safety and satisfaction, it is essential to identify and manage postoperative complications. Despite not being a routine contact, the incidence rate of complications on the seventh and fourteenth postoperative days is noted. According to our investigation, the type of anesthesia, surgical specialty, and duration of surgery should be carefully considered when establishing individualized follow-up plans. These plans, using tools adjusted to the population of each OSU, such as the calculator presented, may allow the available resources to be used with the greatest potential benefit for both patients and healthcare systems.

## Introduction

The long-term financial sustainability of state health systems is a worldwide challenge [1,2]. The growth and evolution of regional anesthesia and minimally invasive techniques went hand in hand with an evidence-based shift to day surgery [3]. Outpatient surgeries are a safe, efficient, and cost-effective alternative to conventional surgery that favors health expenditure rationalization, without compromising patients’ safety and the quality of the service provided [1]. Post-discharge follow-up is an important tool to identify complications whose proper management is crucial for the success of this intervention regimen [3]. It also aims to audit postoperative symptoms and is related to improving quality indicators such as the degree of patient satisfaction [3]. The follow-up phone call is considered an advantageous intervention and is frequently included in European postoperative routines [4-6]. Furthermore, guidelines from the Association of Anaesthetists and the British Association of Day Surgery recommend a helpline for at least the first 24 hours after discharge and a follow-up call the next day [3]. Timing and frequency of follow-up calls should consider the specific characteristics of each patient [4]. This contributes to improving the satisfaction of both patients and their care providers [4]. Also, identifying which calls are useful and improve the quality of care, may optimize human resources management [1]. Several models for personalized follow-up schemes have already been published [6,7]. However, there is currently a lack of scientific evidence to support the development and validation of standardized protocols in this field [4]. In Portugal, follow-up calls are mandatory 24 hours after discharge [8]. In addition, it is also recommended that an extra phone call be made in the following circumstances: on the seventh day after discharge in the elderly, to check specific pre-established conditions; between 48 and 72 hours, and on the seventh day after discharge in case of the neuraxial technique; and at 72 hours post-discharge if a complication was previously identified [9,10]. During the coronavirus disease 2019 (COVID-19) pandemic, the Guiding Standards issued by the Portuguese Outpatient Surgery Association instituted a phone call on the seventh and fourteenth days after discharge for all patients [11].

The aim of our study was to identify complications detected in follow-up calls on the seventh and fourteenth days after discharge in order to develop a model to create an individualized follow-up strategy in our Outpatient Surgery Unit (OSU) in the future.

## Materials and methods

We performed a retrospective, cohort, single-center study from May 8 to July 24, 2020 (a total of 78 days, the period in which both calls were performed to all patients). Patients undergoing surgery at the OSU of the Vila Nova de Gaia/Espinho Hospital Centre (VNGEHC), a tertiary hospital in Portugal, were included. Interventions performed by oral surgeons and anesthesiology were the only ones excluded due to the reduced number of observations [12]. The data were obtained by analyzing clinical records regarding follow-up calls and the respective ambulatory surgeries. The variables analyzed were sex, age, surgical specialty, anesthetic technique, physical status classification as established by the American Society of Anesthesiologists (ASA), surgery duration, and complications registered in the follow-up calls performed seven and 14 days after discharge. All patient complaints identified in the follow-up call were considered relevant and categorized as complications. Statistical analysis was performed with Statistical Product and Service Solutions (SPSS) software version 27.0 (IBM Corp., Armonk, New York). Categorical variables are presented as frequencies and percentages, and continuous variables as means and standard deviations (SD) or medians and interquartile ranges (IQR) for variables with skewed distributions. The different variables under analysis were compared between the groups with and without complications identified in the follow-up calls. For nominal variables, the chi-squared test was used. The independent sample t-test was used for quantitative variables with normal distribution, and the Mann-Whitney U test was used for quantitative variables with skewed distribution. Levene's test assessed the equality of variances between the two groups when analyzing quantitative variables. All reported p-values are two-tailed, with a p-value of 0.05 indicating statistical significance. Binary logistic regressions were adjusted using a stepwise backward-likelihood ratio elimination procedure, considering detecting complications in the follow-up calls as dependent variables. The other analyzed variables were included as predictors if statistical significance was identified in the univariate analysis. The absence of anesthesiology support and ASA I physical status classification were considered reference categories. For variables without a clinically justified comparison group, the category with the most significant number of elements was used as a reference category. The predictive performance of the models was tested by assessing their discrimination (correct classification) and calibration (whether probabilities predicted by the model matched observed probabilities). The Hosmer-Lemeshow test was used to measure the quality of the models' fit and as a proxy for their calibration. The predictors included in the final models are presented as odds ratios (OR), including 95% confidence intervals (CI).

The study was performed according to the Declaration of Helsinki and approved by the ethics committee of VNGEHC (approval number 57/2021).

## Results

Participants

The study included 984 consecutive patients (mean age 50 ± 17 years, 58.1% females, n=572). Age ranged between four and 93 years. Most patients were classified as physical status ASA II (n = 790, 80.3%), and the median duration of procedures was 28 minutes (IQR 28 minutes). Considering the total number of cases, 79.8% (n=785) and 75.3% (n=741) answered the follow-up calls, seven and 14 days after discharge, respectively. No cases of severe acute respiratory syndrome coronavirus 2 (SARS-CoV-2) infection were identified. The univariate analysis for each call, comparing the groups with and without complications, is shown in Table 1 and Table 2.

**Table 1:** Univariate analysis regarding the follow-up call seven days after discharge ASA - American Society of Anesthesiologists physical status classification; IQR – interquartile range; n - number of cases; SD - standard deviation

	Univariate analysis regarding the follow-up call seven days after discharge (n=785)						
Variables	All		Complications				p-value
			Yes		No		
	(n=785)		(n=370)		(n=415)		
Age (years) - mean ± SD	49.8	±17.1	48.9	±17.8	50.7	±16.5	0.137
Sex - n (%)							0.002
Male	332	(42.3)	178	(48.1)	154	(37.1)	-
Female	453	(57.7)	192	(51.9)	261	(62.9)	-
ASA - n (%)							0.524
I	128	(16.3)	62	(16.8)	66	(15.9)	-
II	625	(79.6)	296	(80.0)	329	(79.3)	-
III	32	(4.1)	12	(3.2)	20	(4.8)	-
Anesthesia - n (%)							<0.001
No Anesthesiology support	140	(17.8)	45	(12.2)	95	(22.9)	-
General anesthesia	189	(24.1)	105	(28.4)	84	(20.2)	-
Regional anesthesia	191	(24.3)	101	(27.3)	90	(21.7)	-
Monitored anesthetic care	265	(33.8)	119	(32.2)	146	(35.2)	-
Surgical Specialty - n (%)							<0.001
Plastic surgery	101	(12.9)	34	(9.2)	67	(16.1)	-
General surgery	128	(16.3)	79	(21.4)	49	(11.8)	-
Vascular surgery	157	(20.0)	71	(19.2)	86	(20.7)	-
Gynecology	79	(10.1)	23	(6.2)	56	(13.5)	-
Neurosurgery	48	(6.1)	30	(8.1)	18	(4.3)	-
Orthopedics	130	(16.6)	60	(16.2)	70	(16.9)	-
Otorhinolaryngology	48	(6.1)	19	(5.1)	29	(7.0)	-
Urology	94	(12.0)	54	(14.6)	40	(9.6)	-
Surgery duration (minutes) - median (IQR)	30	(28)	31	(30)	25	(27)	<0.001

**Table 2:** Univariate analysis regarding the follow-up call 14 days after discharge ASA - American Society of Anesthesiologists physical status classification; IQR - interquartile range; n – number of cases; SD - standard deviation

	Univariate analysis regarding the follow-up call 14 days after discharge (n=741).						
Variables	All		Complications				p-value
			Yes		No		
	(n=741)		(n=221)		(n=520)		
Age (years) - mean ± SD	50.6	±16.4	51.7	±14.6	50.1	±17.1	0.200
Sex - n (%)							0.641
Male	308	(41.6)	89	(40.3)	219	(42.1)	-
Female	433	(58.4)	132	(59.7)	301	(57.9)	-
ASA - n (%)							0.026
I	104	(14.0)	24	(10.9)	80	(15.4)	-
II	607	(81.9)	193	(87.3)	414	(79.6)	-
III	30	(4.0)	4	(1.8)	26	(5.0)	-
Anesthesia - n (%)							<0.001
No Anesthesiology support	125	(16.9)	15	(6.8)	110	(21.2)	-
General anesthesia	175	(23.6)	61	(27.6)	114	(21.9)	-
Regional anesthesia	196	(26.5)	69	(31.2)	127	(24.4)	-
Monitored anesthetic care	245	(33.1)	76	(34.4)	169	(32.5)	-
Surgical Specialty - n (%)							<0.001
Plastic surgery	85	(11.5)	10	(4.5)	75	(14.4)	-
General surgery	120	(16.2)	50	(22.6)	70	(13.5)	-
Vascular surgery	161	(21.7)	58	(26.2)	103	(19.8)	-
Gynecology	75	(10.1)	9	(4.1)	66	(12.7)	-
Neurosurgery	48	(6.5)	26	(11.8)	22	(4.2)	-
Orthopedics	131	(17.7)	46	(20.8)	85	(16.3)	-
Otorhinolaryngology	46	(6.2)	3	(1.4)	43	(8.3)	-
Urology	75	(10.1)	19	(8.6)	56	(10.8)	-
Surgery duration (minutes) - median (IQR)	30	(30)	30	(32)	28	(27)	0.266

Complications

Clinical intercurrences were reported in 47.1% (n=370) and 29.8% (n=221) of the follow-up calls answered seven and 14 days after discharge, respectively. The complication with the highest incidence rate was pain, recorded in 44.7% (n=351, six of which severe - numerical scale (NS) 7-10/10) and 26.6% (n=197, four of which severe) of calls answered on the 7th and 14th days after discharge, respectively. In most cases, the pain was classified as mild (NS 1-3/10), and there was an overall reduction in its incidence rate in the second phone call. Other complications registered in the first call include hematoma (n=4, 0.5%), hematic (n=6, 0.8%), or purulent (n=5, 0.6%) drainage at the surgical site, post-dural puncture headache (n=4, 0.5%) and nausea/vomiting (n=4, 0.5%). In the second call, there was an increase in the incidence rate of sensory disorders without functional impairment (n=7, 0.9%), blood drainage at the surgical site (n=6, 0.8%), and suture dehiscence (n=5, 0.7%). Additional information regarding the identified complications is presented in Table 3.

**Table 3:** Incidence of complications registered in the follow-up call ^ⱡ ^- Multiple follow-up calls recorded several complications; DP - dural puncture; FI - functional impairment; n – number of complications recorded; NS - numerical scale

	Incidence of complications registered in the follow-up call (the incidence rate is presented in brackets).			
Complication	Follow-up call			
	7^th^ day after discharge		14^th^ day after discharge	
	(n=384^ⱡ^)		(n=223^ⱡ^)	
Mild pain (NS 1-3/10)	314	(40.00)	179	(24.16)
Moderate pain (NS 4-6/10)	31	(3.95)	14	(1.89)
Intense pain (NS 7-10/10)	6	(0.76)	4	(0.54)
Sensory disorder (no FI)	3	(0.38)	7	(0.94)
Sensory disorder (with FI)	2	(0.25)	1	(0.13)
Nausea/vomiting	4	(0.51)	0	(0.00)
Headache (without DP)	1	(0.13)	1	(0.13)
Post-DP headache	4	(0.51)	1	(0.13)
Suture dehiscence	2	(0.25)	5	(0.67)
Purulent drainage (surgical site)	5	(0.64)	4	(0.54)
Blood drainage (surgical site)	6	(0.76)	6	(0.81)
Bruise (surgical site)	4	(0.51)	1	(0.13)
Urinary retention	2	(0.25)	0	(0.00)

Follow-up call seven days after discharge

Regarding the phone call seven days after discharge, there were significant statistical differences between the two groups in all the variables analyzed, except for age (p=0.137) and ASA physical status classification (p=0.524). In the multivariate analysis performed to create the binary logistic regression model, only the type of anesthesia (p=0.004), surgical specialty (p<0.001), and surgery duration (p=0.004) remained independent risk factors for detecting complications in this call (Table 4).

**Table 4:** Binary logistic regression for detecting complications in the follow-up call seven days after discharge CI - confidence interval; OR - odds ratio

	Binary logistic regression for detecting complications in the follow-up call seven days after discharge.	
Independent variables	OR	95% CI for OR
Anesthesia	-	-
No anesthesiology support	1	-
General anesthesia	2.52	1.52 to 4.18
Regional anesthesia	2.04	1.21 to 3.43
Monitored anesthetic care	1.75	1.10 to 2.80
Surgical Specialty	-	-
Vascular surgery	1	-
General surgery	2.08	1.26 to 3.43
Plastic surgery	1.05	0.58 to 1.90
Gynecology	0.82	0.43 to 1.56
Neurosurgery	3.60	1.70 to 7.61
Orthopedics	1.37	0.82 to 2.31
Otorhinolaryngology	1.05	0.52 to 2.15
Urology	2.69	1.51 to 4.80
Surgery duration (minutes)	1.01	1.00 to 1.02

In the group of patients undergoing procedures without anesthesiology support, complications were detected in 32.1% of cases (n=45). Compared to this group, patients undergoing procedures under general or regional anesthesia were 2.52 (p<0.001; 95% CI 1.52-4.18) and 2.04 (p=0.007; 95% CI 1.21-3.43) times more likely to have complications detected in this call, respectively. Also, patients undergoing monitored anesthetic care were 1.75 (p=0.019; 95% CI 1.10-2.80) times more likely to have complications detected. Complications were detected in 45.2% (n=71) of patients undergoing vascular surgery procedures. Compared with these, patients undergoing general surgery, neurosurgery, or urology procedures were more than twice as likely to have complications registered in this call. An analogous interpretation for the remaining cases is unreliable since the OR's CI includes one. Each minute increase in surgery duration increased the odds of complications by 1.1% (p=0.004; 95% CI 1.003-1.018). The Hosmer-Lemeshow test p-value was 0.102, so the model created is considered to have an appropriate calibration [13]. The overall classification accuracy of the model was 62.0%.

Follow-up call 14 days after discharge

Regarding the call 14 days after discharge, there were significant statistical differences between the two groups for ASA physical status classification (p=0.026), type of anesthesia (p<0.001), and surgical specialty (p<0.001). In the multivariate analysis, only the type of anesthesia (p=0.004) and the surgical specialty (p<0.001) remained independent risk factors for complications in this call (Table 5). 

**Table 5:** Binary logistic regression for detecting complications in the follow-up call 14 days after discharge ASA - American Society of Anesthesiologists physical status classification; CI - confidence interval; OR - odds ratio

	Binary logistic regression for detecting complications in the follow-up call 14 days after discharge.	
Independent variables	OR	95% CI for OR
Anesthesia	-	-
No anesthesiology support	1	-
General anesthesia	3.21	1.57 to 6.55
Regional anesthesia	2.36	1.17 to 4.77
Monitored anesthetic care	3.11	1.60 to 6.05
Surgical Specialty	-	-
Vascular surgery	1	-
General surgery	1.01	0.61 to 1.68
Plastic surgery	0.22	0.10 to 0.50
Gynecology	0.22	0.10 to 0.49
Neurosurgery	2.09	0.98 to 4.46
Orthopedics	0.78	0.46 to 1.33
Otorhinolaryngology	0.10	0.03 to 0.34
Urology	0.63	0.32 to 1.23
ASA	-	-
I	1	-
II	0.93	0.54 to 1.60
III	0.40	0.12 to 1.37

The ASA physical status classification (p=0.324) was also included in the binary logistic regression model, as it was associated with a significant increase in its calibration (Hosmer-Lemeshow test p-value 0.424 versus 0.091). In the group of patients undergoing procedures without anesthesiology support, complications were registered in 12.0% of cases (n=15). Compared to this group, patients undergoing procedures under general anesthesia or monitored anesthetic care are 3.21 (p=0.001; 95% CI 1.57-6.55) and 3.11 (p<0.001; 95% CI 1.60-6.05) times more likely to have complications detected, respectively. Patients undergoing regional anesthesia are 2.36 (p=0.017; 95% CI 1.17-4.77) times more likely to have complications registered. Complications were detected in 36.0% (n=58) of patients undergoing vascular surgery procedures. Compared to these, patients undergoing procedures in the field of plastic surgery, gynecology, or otorhinolaryngology are less likely to have complications detected in this call (p<0.001 in all three cases; OR 95% CI 0.10-0.50; 0.10-0.49; 0.03-0.34, respectively). The Hosmer-Lemeshow test p-value was 0.424, so the model created is considered to have appropriate calibration [13]. The overall classification accuracy of the model was 71.3%.

Calculator model

From the regression models performed, a calculator was created to predict the detection of complications in the follow-up calls made seven and 14 days after discharge, which is available at the following link: https://calculatormodel.wordpress.com/ [14].

## Discussion

Health resources rationalization is required in universal health coverage [1,2]. It allows health professional's allocation for tasks that effectively contribute to improving healthcare quality and favors the hospital's financial sustainability [1,2]. Outpatient surgery has been expanding steadily, allowing procedures to be carried out safely, efficiently, and cost-effectively [6,15]. It represents a relevant approach for health cost rationalization with benefits for the patient [1,15]. To optimize the outcomes, it is important to monitor results as a tool for auditing strategies [4]. Therefore, based on the data collected, we aimed to develop a personalized model that can be used for individualized follow-up strategies of our OSU. We identified factors with a statistically significant impact on the occurrence of complications detected in the follow-up calls performed seven and 14 days after discharge in our OSU (type of anesthesia, surgical specialty, and duration of surgery). Then, a calculator model that allows estimating the probability of complications identified was built. We believe that the model created can be integrated into the overall analysis of the patient before hospital discharge, to establish an individualized follow-up plan. Therefore, in this study, we believe that calibration is more relevant than the discriminatory capacity of the models. To our knowledge, there are no published analogous studies. The type of anesthesia and surgical specialty was found to be independent risk factors for the detection of complications in the phone calls under analysis at our OSU. Not surprisingly, less invasive and complex procedures are generally performed without anesthesiology support and a lower rate of complications in follow-up calls is reported. Furthermore, monitored anesthetic care was associated with a lower incidence of complications when compared to general (the group of patients with the highest rate of complications) and regional anesthesia in the first phone call. The use of regional techniques seems to be a protective factor in the occurrence of complications detected on the 14th day post-discharge call in our population and is associated with a lower rate of complications compared to the group of patients receiving general anesthesia or monitored anesthetic care, whose complications odds are close. The most frequent complication is pain, and regional techniques are known to effectively contribute to minimizing it, as part of multimodal analgesia [9]. Besides, regional anesthesia provides anesthesia for the procedure, prolonged analgesia, and faster oral intake with earlier hospital discharges [9]. With a bigger sample, it would be interesting to compare complications reported in patients submitted to the same surgical procedures under general versus regional anesthesia, to enhance and adjust the model created. The duration of surgery had a particularly relevant impact on the occurrence of complications seven days after discharge, being an independent risk factor for complications. This corroborates the importance of a well-done selection of patients and procedures for day surgery [16]. Although surgery duration has already been considered a risk factor for adverse outcomes in outpatient surgery, it is worth noting that in our study an increase of only one minute in surgery duration, increased the odds of complications by 1.1% [17]. A similar relation has been described previously in a meta-analysis, which also included inpatient surgeries [18]. The ASA physical status also appears to influence the outcomes. Contrary to what would be expected and is described in the literature, patients classified as ASA III appear to have a lower detection of complications in the second phone call under analysis [19,20]. We believe this may result from the admission criteria at our OSU. These patients are predominantly accepted to less complex and invasive procedures, ensuring they are clinically stable during the preoperative period. In our OSU, different surgical specialties perform procedures with varying degrees of complexity, which we believe contributes to the different incidences of complications detected for each surgical specialty. According to our results, patients' age and sex do not seem to have a significant impact on the occurrence of complications in the follow-up calls performed seven and 14 days after discharge. In fact, evidence shows that patients' comorbidities are more relevant than their age [16]. The type and incidence of complications detected are consistent with other published studies [21,22]. We recognize the importance of a comprehensive analysis to implement measures that reduce their incidence. We believe that an individualized follow-up plan, adjusted to them, can also audit their evolution. Additionally, and considering Portuguese guidelines, no recommendations for phone calls after the seventh day are present although complications were identified during that period. This should draw attention to at-risk patients, whose follow-up period could be extended. Besides, complications were reported on the fourteenth day, although some of these patients reported no events on the first phone call. Without this second contact, some complications would be overlooked. We believe that the development of a tool adapted to the specificities of our OSU population, types of procedures, and stakeholders could contribute to resource rationalization. We consider that the incorporation of the model presented in the development of individualized follow-up plans can contribute to improving the management of post-discharge complications, through its identification and early approach. This can contribute to improving the quality of patient care with a more effective rationalization of available resources.

Limitations

We identified some limitations in our study worth mentioning. Being a single-center study, the data are of limited applicability. We recognize the diversity of population and surgical interventions in each OSU, so we emphasize the need to adapt the data presented to each unit and encourage the development of similar studies to create calculators adjusted to it. We also recognize the need to regularly update the data incorporated in the model created, so that it remains adjusted to the specificities of patients and the evolution of procedures, techniques, and teams. The second limitation of this study is the number and types of variables. Due to the predicted sample size, the inclusion of a high number of variables or multiple variables with a high number of categories would prevent its incorporation in the created model, once lower values of events per variable have often been associated with poorer predictive performance after the validation of the developed models [23]. Thus, we recognize the need for future studies with a larger sample size that includes additional variables considered relevant in this area. Past medical history and specific surgical procedures within each specialty may be considered [17]. With larger samples, it could be useful to distinguish different categories of complications. Also, the information analyzed was collected from previous records, so registration errors cannot be excluded. Finally, we recognized the need for larger investigations to validate the calculator presented, as well as to increase its accuracy and calibration.

## Conclusions

To guarantee the quality of work and patient safety and satisfaction, promptly identifying and managing postoperative complications is essential, particularly in an outpatient surgery regimen. Despite not being a routine contact, the incidence rate of complications on the seventh and fourteenth postoperative days is not negligible, with pain being the most frequent. Individualized follow-up plans, using tools adjusted to the population of each OSU, such as the calculator presented, may allow the available resources to be used in patients with the greatest potential benefit from these additional contacts. According to the reality of our OSU and the follow-up calls analyzed, the type of anesthesia, surgical specialty, and duration of surgery should be carefully considered when establishing individualized follow-up plans. Further studies, with a larger sample size that includes additional variables considered relevant in this area, are needed.

## References

[1] Matos R, Ferreira D, Pedro MI (2021). Economic analysis of Portuguese public hospitals through the construction of quality, efficiency, access, and financial related composite indicators. Soc Indic Res.

[2] Keliddar I, Mosadeghrad AM, Jafari-Sirizi M (2017). Rationing in health systems: a critical review. Med J Islam Repub Iran.

[3] Bailey CR, Ahuja M, Bartholomew K (2019). Guidelines for day-case surgery 2019: guidelines from the Association of Anaesthetists and the British Association of Day Surgery. Anaesthesia.

[4] Johnson M, Laderman M, Coleman E (2013). Enhancing the effectiveness of follow-up phone calls to improve transitions in care: three decision points. Jt Comm J Qual Patient Saf.

[5] Daniels SA, Kelly A, Bachand D, Simeoni E, Hall C, Hofer SM, Hayashi A (2016). Call to care: the impact of 24-hour postdischarge telephone follow-up in the treatment of surgical day care patients. Am J Surg.

[6] Dahlberg K, Jaensson M, Nilsson U (2019). "Let the patient decide" - person-centered postoperative follow-up contacts, initiated via a phone app after day surgery: secondary analysis of a randomized controlled trial. Int J Surg.

[7] Forster A, LaBranche R, McKim R (2008). Automated patient assessments after outpatient surgery using an interactive voice response system. Am J Manag Care.

[8] Secretário de Estado Adjunto e da Saúde - Ministério da Saúde. Despacho n.o 30114/2008 (2022). Dispatch 30114/2008, of November 21st [In Portuguese]. https://dre.tretas.org/dre/242841/despacho-30114-2008-de-21-de-novembro.

[9] (2022). Recommendations for regional anesthesia in outpatient surgery [In Portuguese]. Assoc Port Cir Ambulatória. Published online.

[10] Vieira V, Carmona C, Pinto J, Marcos A (2017). Recommendations for the anesthetic approach to the elderly patient in ambulatory surgery [Article in Portuguese]. Rev Soc Port Anestesiol.

[11] Associação Portuguesa de Cirurgia de Ambulatório (2020). National Recommendations: Return of Surgical Activity in the Covid-19 Era. Cirurgia de Ambulatório [Book in Portuguese]. Recom Nac • Retorno Da Atividade Cirúrgica Na Era Covid-19 Cir Ambulatório. Published online.

[12] Kroonenberg P, Verbeek A (2018). The tale of Cochran’s rule: my contingency table has so many expected values smaller than 5, what am I to do?. Am Stat.

[13] Huang Y, Li W, Macheret F, Gabriel RA, Ohno-Machado L (2020). A tutorial on calibration measurements and calibration models for clinical prediction models. J Am Med Inform Assoc.

[14] (2022). Calculator model. https://calculatormodel.wordpress.com/.

[15] Jaensson M, Dahlberg K, Nilsson U (2019). Factors influencing day surgery patients' quality of postoperative recovery and satisfaction with recovery: a narrative review. Perioper Med (Lond).

[16] Rajan N, Rosero EB, Joshi GP (2021). Patient selection for adult ambulatory surgery: a narrative review. Anesth Analg.

[17] Pinto F, Irwin M (2022). Patient selection for day surgery. Anaesth Intensive Care Med.

[18] Cheng H, Clymer JW, Po-Han Chen B, Sadeghirad B, Ferko NC, Cameron CG, Hinoul P (2018). Prolonged operative duration is associated with complications: a systematic review and meta-analysis. J Surg Res.

[19] Foley C, Kendall MC, Apruzzese P, De Oliveira GS (2021). American Society of Anesthesiologists Physical Status Classification as a reliable predictor of postoperative medical complications and mortality following ambulatory surgery: an analysis of 2,089,830 ACS-NSQIP outpatient cases. BMC Surg.

[20] Ansell GL, Montgomery JE (2004). Outcome of ASA III patients undergoing day case surgery. Br J Anaesth.

[21] Goldfarb CA, Bansal A, Brophy RH (2017). Ambulatory surgical centers: a review of complications and adverse events. J Am Acad Orthop Surg.

[22] Mihailescu SD, Maréchal I, Thillard D, Gillibert A, Compère V (2020). Socioenvironmental criteria and postoperative complications in ambulatory surgery in a French university hospital: a prospective cross-sectional observational study. BMJ Open.

[23] van Smeden M, Moons KG, de Groot JA, Collins GS, Altman DG, Eijkemans MJ, Reitsma JB (2019). Sample size for binary logistic prediction models: beyond events per variable criteria. Stat Methods Med Res.

